# The expression changes of PD-L1 and immune response mediators are related to the severity of primary bone tumors

**DOI:** 10.1038/s41598-023-47996-8

**Published:** 2023-11-22

**Authors:** Amir Reza Eghtedari, Mohammad Amin Vaezi, Elaheh Safari, Vahid Salimi, Banafsheh Safizadeh, Pegah Babaheidarian, Amene Abiri, Elmira Mahdinia, Alireza Mirzaei, Parisa Mokhles, Masoumeh Tavakoli-Yaraki

**Affiliations:** 1 Department of Biochemistry, School of Medicine, Iran University of Medical Sciences, P.O. Box: 1449614535, Tehran, Iran; 2 Department of Immunology, School of Medicine, Iran University of Medical Sciences, Tehran, Iran; 3 Department of Virology, School of Public Health, Tehran University of Medical Sciences, Tehran, Iran; 4 Department of Pathology, School of Medicine, Iran University of Medical Sciences, Tehran, Iran; 5 Department of Obstetrics and Gynecology, Tehran University of Medical Sciences, Tehran, Iran; 6 Bone and Joint Reconstruction Research Center, Shafa Orthopedic Hospital, Iran University of Medical Sciences, Tehran, Iran; 7 School of Medicine, Iran University of Medical Sciences, Tehran, Iran

**Keywords:** Cancer, Molecular biology, Oncology, Pathogenesis

## Abstract

The expression pattern, diagnostic value, and association of PD-L1, IFN-γ and TGF-β with bone tumor type, severity, and relapse are determined in this study. 300 human samples from patients with osteosarcoma, Ewing sarcoma, and GCT were enrolled. The PD-L1 gene and protein expression were assessed by qRT-PCR and immunohistochemistry, respectively. ELISA and flow cytometry was used to detect cytokines and CD4/CD8 T cell percentages, respectively. A considerable increase in PD-L1 level was detected in bone tumor tissues at both gene and protein levels that was considerable in osteosarcoma and Ewing sarcoma. A positive correlation was detected regarding the PD-L1 and tumor metastasis and recurrence in osteosarcoma and Ewing sarcoma. The increased IFN-γ level was detected in patients with metastatic, and recurrent osteosarcoma tumors that were in accordance with the level of TGF-β in these samples. The simultaneous elevation of IFN-γ and TGF-β was detected in Ewing sarcoma and GCT, also the CD4 + /CD8 + ratio was decreased significantly in patients with osteosarcoma compared to GCT tumors. The elevated levels of PD-L1, TGF- β, and IFN-γ were associated with bone tumor severity that can provide insights into the possible role of this axis in promoting immune system escape, suppression, and tumor invasion.

## Introduction

Sarcomas are rare heterogeneous malignancies of mesenchymal origin that mostly develop in bone and connective tissues and account for 1% of all cancer diagnoses in adults^1^. Primary bone tumors are classified as sarcomas and characterized by high morbidity and mortality with diverse genetically and histologically malignant and benign subtypes^2^. Osteosarcoma is the most prevalent form of bone cancer, primarily impacting the cells responsible for creating new bone tissue. These tumors originate from osteoblasts and typically develop in the long bones and are known for their aggressive nature^3^. Osteosarcoma tumors consist of malignant cells that produce abnormal bone matrix and their level of differentiation is often used to determine their grade^4^. Ewing sarcoma is a rare and malignant type of primary bone tumor that mainly arises in the bones or soft tissues. It commonly occurs in the long bones of the arms and legs, pelvis, chest wall, or spine^5^. These tumors originate from neuroectodermal cells, which are primitive nerve cells. The majority of Ewing sarcomas are characterized by a specific genetic abnormality called a translocation between chromosomes 11 and 22. This translocation causes the fusion of two genes, EWSR1 on chromosome 22 and FLI1 on chromosome 11 that results in production of an abnormal protein that promote the growth of cancer^6^. Giant cell tumors (GCTs) of bone are rare tumors that can be intermediate (rarely-metastasizing) or malignant^7^. They primarily affect the bones, with a higher occurrence in the long bones^8^. These tumors are characterized by the presence of numerous multinucleated giant cells surrounded by mononuclear stromal cells. Although most giant cell tumors are rarely metastasizing, they can still lead to significant complications and require appropriate management^9^. Despite the fact that chemotherapy-based treatments besides wide surgery developed the outcome of this disease and five-year survival, the postoperative recurrence rates of these tumors are still considerable and the curative rate of metastatic lesions is not satisfactory^10,11^. It is postulated that immune suppression, immune escape, and tumor immune microenvironment play pivotal roles in the rate of patients' response to chemotherapy and the possibility of tumor recurrence^12,13^. Immune suppression is driven by inhibiting the functions of T cells and the subsequent immune system impairment caused by aberrant expression of immune checkpoint molecules^14^. The programmed death 1 (PD-1) and programmed death ligand1 (PD-L1) are known as immune checkpoints that contribute to regulating immune tolerance and surveillance within the tumor microenvironment^15^, T cell proliferation and activation^16^ also anti-tumor immune response^17^. PD-1 is a transmembrane protein that is expressed by diverse immune system components such as activated T cells, natural killer (NK) cells, and B lymphocytes. On the other hand, PD-L1 is a type 1 transmembrane glycoprotein that is mainly expressed by activated T cells as well as macrophages and epithelial cells^15^. Of note, PD-L1 is reported to be expressed on the surface of tumor cells and following binding to its receptor on the surface of cytotoxic T cells, the tumor cell can escape immune surveillance and mediate activation of proliferative and survival signaling pathways^18^. Besides, PD-L1 can also exert non-immune proliferative effects through the induction of mesenchymal phenotype to facilitate tumor cell invasion and cancer progression^19^. The wide range of effects following PD-L1: PD-1 interaction indicates that multiple intrinsic signaling pathways may involve in the regulation of this axis. It has been demonstrated that interferon γ (IFN-γ) induces over-expression of PD-L1 by tumor cells through manipulation of various signaling transducers such as protein kinase D isoform 2 (PKD2), the Janus kinase (JAK)1, JAK2 and signal transducer and activator of transcription (STAT)1 pathway^20^. IFN-γ is a pleiotropic cytokine that plays a critical role in orchestrating both innate and adaptive immune response and regulating pro-tumorigenic or anti-tumorigenic responses in the tumor microenvironment^21^. Moreover, the immune system homeostasis and the biological function of tumor cells can be influenced by the transforming growth factor-beta (TGF-β) family of cytokines that regulate the stemness feature of tumor cells and the level of response to immunotherapies^22^. It was shown that up-regulation of TGF- β in cancer caused T cell and NK cells suppression and regulatory T cells (Tregs) elevation leading to immunosuppressive effects^23^. These findings have improved our understanding of the importance and complexity of immune checkpoints and their modulators in tumor fate and biology. Based on these perspectives, the present study is aimed to determine: (1) the expression profile of PD-L1 in most prevalent primary bone tumors, (2) the expression level of IFN-γ, as a well-described modulator of PD-L1: PD-1 checkpoint, (3) the circulating level of TGF- β in primary bone tumors; (4) the association of PD-L1, IFN-γ, and TGF- β aberrant expression with primary bone tumor severity, metastasis and recurrence; and (5) the relevance of PD-L1, IFN-γ and TGF- β expression profile with the status of CD4/CD8 in patients with malignant and benign bone tumors.

## Results

### The PD-L1 gene expression level increased in patients with primary bone tumors

**Figure 1:**
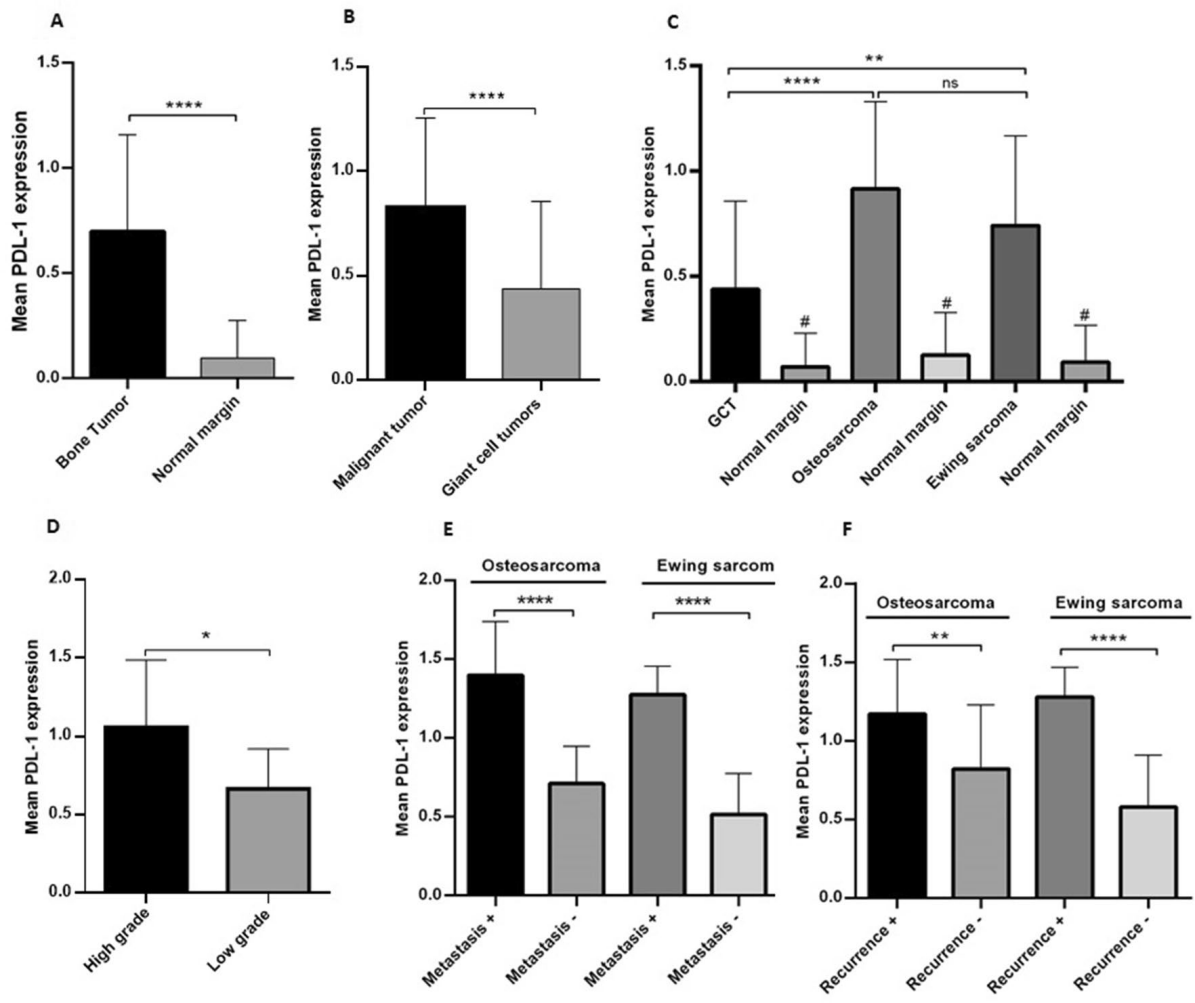
The gene expression pattern of PD-L1 in primary bone tumor tissue, non-cancerous bone tissue and tumor subtypes. An elevated level of PD-L1 mRNA expression was detected in in bone tumor tissues compared to the noncancerous tissues (**A**), also malignant tumors compared to GCT (**B**). All three tumors types expressed higher level of PD-L1 compared to their matched tumor margins; also osteosarcoma and Ewing sarcoma tumors over-expressed PD-L1 compared to GCT group (**C**). The expression pattern of PD-L1 in high and low-grade osteosarcoma (**D**), metastatic and non-metastatic (**E**) and recurrent and non-recurrent (**F**) osteosarcoma and Ewing sarcoma is illustrated. The statistical differences between groups are shown as asterisks (* = *P* < 0.05, ** = *P* < 0.01, **** = *P* < 0.0001), (ns) indicates unspecific, (#) indicates *P* < 0.0001 for comparing osteosarcoma, Ewing sarcoma and GCT group with their matched adjacent noncancerous tissues, separately.

Based on the data, the expression level of PD-L1 was significantly increased in bone tumors compared to non-cancerous tumor margin (*P* < 0.0001) with the mean and standard error of the mean (SEM) of 0.69 ± 0.04 for tumor tissues and 0.09 ± 0.01 for tumor margins (Fig. 1A). Additionally, malignant bone tumors (0.82 ± 0.05) expressed higher levels of PD-L1 compared to GCT (0.43 ± 0.07) (*P* < 0.0001) (Fig. 1B). The lowest level of PD-L1 expression was detected in GCT (0.43 ± 0.07) compared to osteosarcoma tumors (0.91 ± 0.07) (*P* < 0.0001) and Ewing sarcoma (0.74 ± 0.07) (*P* = 0.0017) tumors; while the difference between osteosarcoma and Ewing sarcoma tumors was not statistically significant (*P* = 0.09). The above comparison showed a significant increase between all types of tumors compared to the corresponding normal margin (*P* < 0.0001) (Fig. 1C). The PD-L1 expression level was significantly higher in high-grade osteosarcoma tumors (1.05 ± 0.09) compared to low-grade tumors (0.66 ± 0.07) (*P* = 0.015 (Fig. 1D). The PD-L1 gene level was higher in metastatic (1.39 ± 0.11) compared to non-metastatic (0.7 ± 0.05) osteosarcoma tumors (*P* < 0.0001), also metastatic (1.27 ± 0.05) compared to non-metastatic (0.51 ± 0.05) Ewing sarcoma tumors (*P* < 0.0001) (Fig. 1E). Moreover, the PD-L1 gene level was increased considerably in recurrent (1.17 ± 0.12) compared to non-recurrent (0.82 ± 0.08) osteosarcoma tumors (*P* = 0.008), also recurrent Ewing sarcoma (1.28 ± 0.08) compared to non-recurrent Ewing sarcoma tumors (0.57 ± 0.06) (*P* < 0.0001) (Fig. 1F).

### The PD-L1 protein level was increased in osteosarcoma, Ewing sarcoma and GCT

**Figure 2:**
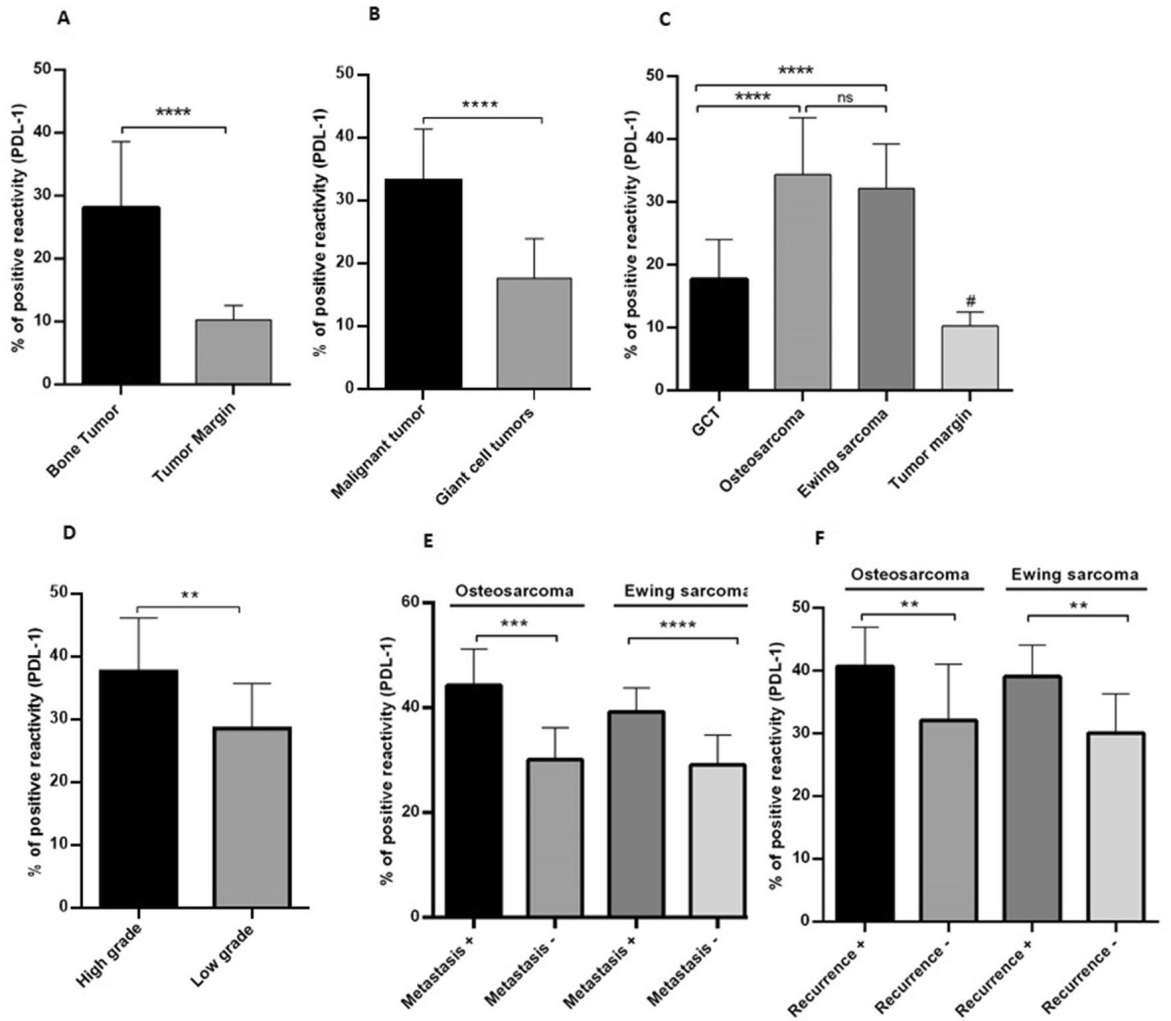
The protein level of PD-L1 in primary bone tumors. The percentage of PD-L1 positive reactivity was increased in tumor tissues compared to non-cancerous tissues (**A**) and malignant tumors compared to GCT (**B**). The elevated level of PD-L1 protein was detected in osteosarcoma and Ewing sarcoma compared to GCT group; while the difference between osteosarcoma and Ewing sarcoma tumors was not significant (**C**). The high-grade osteosarcoma tumors expressed higher PD-L1 protein compared to low -grade counterparts (**D**). Also the metastatic (**E**) and recurrent (**F**) osteosarcoma and Ewing sarcoma tumors showed higher PD-L1 level compared to the non-metastatic and non-recurrent tumors. The statistical differences between groups are shown as asterisks (* = *P* < 0.05, ** = *P* < 0.01, *** = *P* < 0.001, **** = *P* < 0.0001) and (ns) indicates unspecific. (#) indicates *P* < 0.0001 for comparing osteosarcoma, Ewing sarcoma, and GCT with adjacent noncancerous tissues.

**Figure 3:**
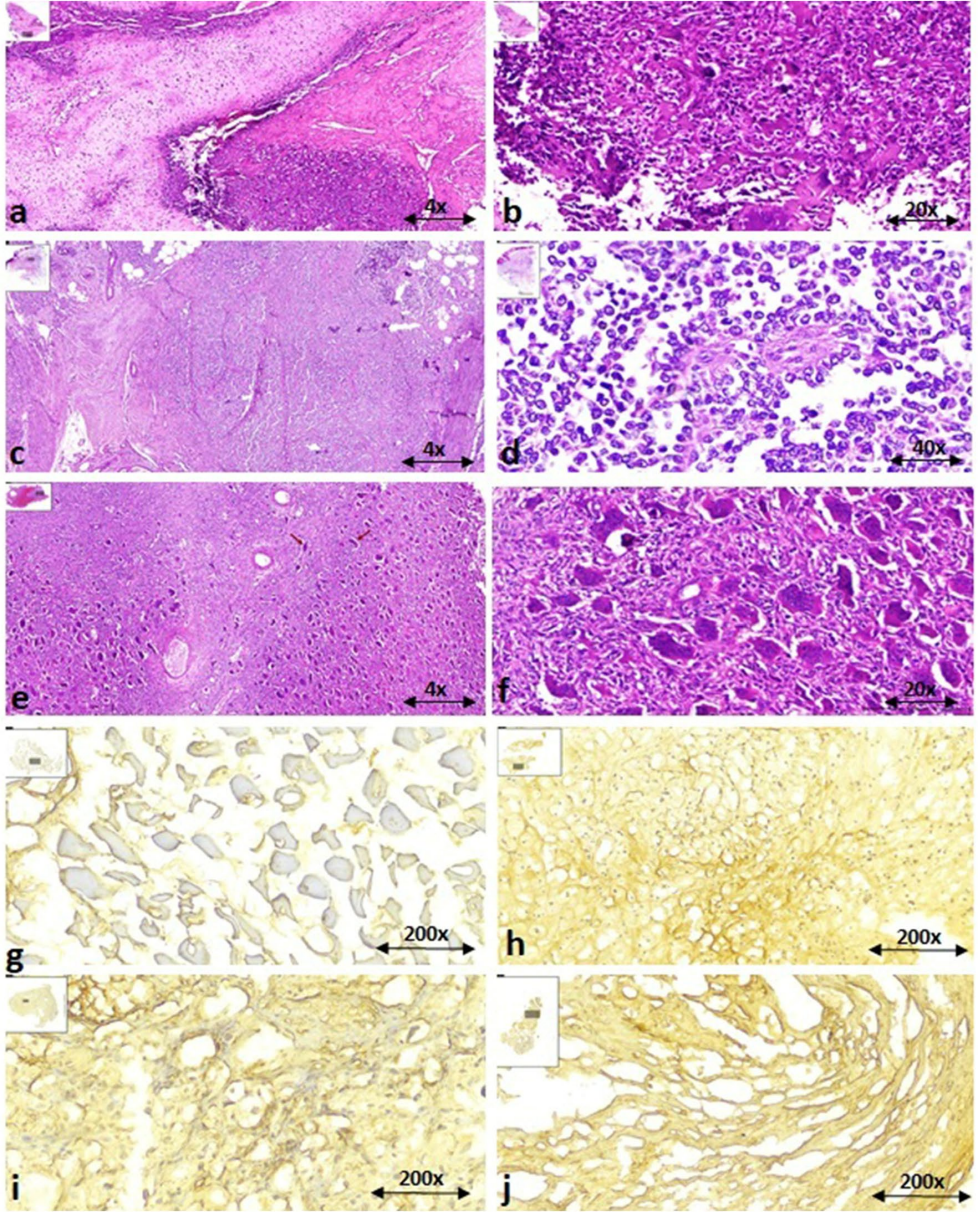
The H&E and immunohistochemistry staining of PD-L1 protein in bone tumors. The representative images for hematoxylin and eosin (H&E) staining of the osteosarcoma tumor tissue (**a**, **b**), Ewing sarcoma (**c**, **d**), and GCT (**e**, **f**) are shown. The negative immune-reactivity of PD-L1 using immunohistochemistry is shown (**g**) and the weak staining intensity (h), the moderate intensity (**i**), and the strong intensity (**j**) of PD-L1 staining are shown. The scale of magnification for each image is shown.

The histopathology of bone tumors was evaluated using hematoxylin and eosin (H&E) staining and the pattern of PD-L1 protein expression in bone tumors was measured using immunohistochemistry. The histopathological analysis of tissue sections from osteosarcoma reveals a densely populated mesenchymal tumor that invades the peripheral soft tissue through the periosteum (Fig. 3a). The neoplastic cells in osteosarcoma display pleomorphism and have ill-defined cytoplasm, arranged within a cartilaginous stroma (Fig. 3b). In the case of Ewing sarcoma, uniform small round cells were detected, infiltrating the adipo-connective tissue diffusely in a sheet-like growth pattern. These cells possess round nuclei, finely stippled chromatin, eosinophilic cytoplasm, and lack distinct cell membranes and form Homer-Wright pseudorosettes (Fig. 3c,d). In the case of giant cell tumor (GCT), the sections exhibit a cellular lesion consisting of numerous multi-nucleated giant cells interspersed with mononuclear cells (Fig. 3e). The giant cells contain centrally located nuclei and exhibit variable morphologies (Fig. 3f). The percentages of positive reactivity of PD-L1 staining intensity in different primary bone tumors are illustrated in Fig. 2 and the representative images of both assays are shown in Fig. 3. Accordingly, it was revealed that the protein level of PD-L1 in bone tumors was increased significantly compared to non-cancerous adjacent tissues (*P* < 0.0001) (Fig. 2A). The higher expression of PD-L1 was detected in malignant tumors compared to GCT (*P* < 0.0001) (Fig. 2B); while the difference between osteosarcoma and Ewing sarcoma tumors was not statistically significant (Fig. 2C). Both osteosarcoma (*P* < 0.0001) and Ewing sarcoma (*P* < 0.0001) tumors showed a higher level of PD-L1 protein level compared to GCT and all the above types of tumors showed increased expression compared to healthy bone tissue (Fig. 2C). Based on data, high-grade osteosarcoma (*P* = 0.002) showed higher intensity of PD-L1 compared to their low-grade counterparts (Fig. 2D). Also, the expression of PD-L1 protein in metastatic osteosarcoma (*P* < 0.0001) and Ewing sarcoma (*P* < 0.0001) tumors was prominent compared to their non-metastatic counterparts (Fig. 2E). Moreover, the recurrent osteosarcoma (*P* = 0.007) and Ewing sarcoma (*P* = 0.0011) tumors showed higher expression of PD-L1 protein compared to the non-recurrent osteosarcoma and Ewing sarcoma tumors, respectively (Fig. 2F).

### The circulating level of IFN-γ enhanced in patients with primary bone tumors

**Figure 4:**
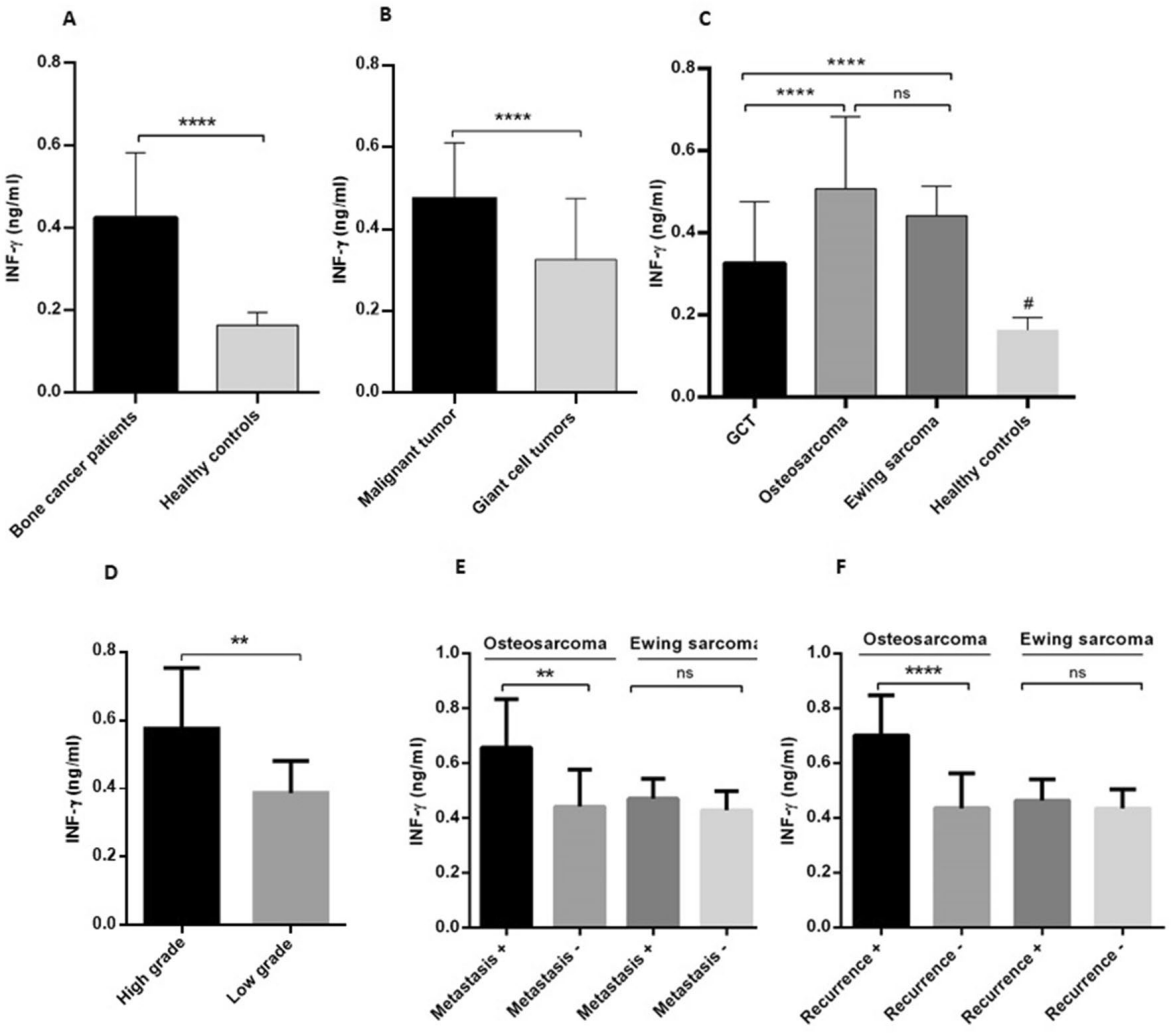
The level of IFN-γ in blood of patients with primary bone tumors and healthy subjects. The IFN-γ level was increased in the blood of patients with bone tumors compared to the healthy subjects (**A**) and patients with malignant tumors compared to GCT (**B**). A significant decrease was detected in patients with GCT compared to patients with osteosarcoma and Ewing sarcoma tumors; while the leveled level of IFN-γ was detected in all types of tumors compared to healthy subjects (**C**). The patients with high-grade osteosarcoma showed higher level of IFN-γ compared to their corresponding opposite groups (**D**). The level of IFN-γ in patients with metastatic (**E**) and recurrent (**F**) osteosarcoma tumors was increased compare to their opposite groups; while the differences in patients with Ewing sarcoma tumors as matter of metastasis and tumor recurrence was nor remarkable. The statistical differences between groups are shown as asterisks (** = *P* < 0.01, *** = *P* < 0.001, **** = *P* < 0.0001), and (ns) indicates unspecific. (#) indicates *P* < 0.0001 for comparing osteosarcoma, Ewing sarcoma, and GCT with healthy subjects.

As it is shown in Fig. 4A, the level of IFN-γ was increased in patients with bone tumors compared to healthy controls (*P* < 0.0001). The mean and SEM of IFN-γ was 0.42 ± 1 = 0.01 in patients and 0.16 ± 0.005 in controls. The malignant (0.47 ± 0.01) bone tumors expressed a higher level of IFN-γ compared to GCT (0.32 ± 0.02) (*P* < 0.0001) (Fig. 4B). The level of IFN-γ was significantly lower in patients with GCT (0.32 ± 0.02) compared to the patients with osteosarcoma (0.50 ± 0.03) and Ewing sarcoma (0.44 ± 0.01) (*P* < 0.0001) tumors; while the difference in the level of IFN-γ between patients with osteosarcoma and Ewing sarcoma was not statistically significant (Fig. 4C). Notably, the level of IFN-γ in all types of primary bone tumors was higher compared to healthy subjects (0.11 ± 0.005) (*P* < 0.0001) (Fig. 4C). Moreover, the patients with high-grade osteosarcoma (0.57 ± 0.04) expressed a higher level of IFN-γ compared to the low -grade osteosarcoma (0.38 ± 0.02) (*P* = 0.003) (Fig. 4D). Patients with metastatic osteosarcoma tumors (0.65 ± 0.05) over-expressed IFN-γ compared to their non-metastatic counterparts (0.44 ± 0.02) (*P* = 0.001); while the difference in patients with Ewing sarcoma tumors as a matter of metastasis was not remarkable (Fig. 4E). Accordingly, patients with recurrent osteosarcoma tumors (0.7 ± 0.05) expressed a higher level of IFN-γ compared to their non-recurrent counterparts (0.43 ± 0.02) (*P* < 0.0001); while the difference between patients with recurrent and non-recurrent Ewing sarcoma tumors was not statistically significant (Fig. 4F).

### The increasing level of TGF- β in patients with osteosarcoma, Ewing sarcoma and GCT

**Figure 5:**
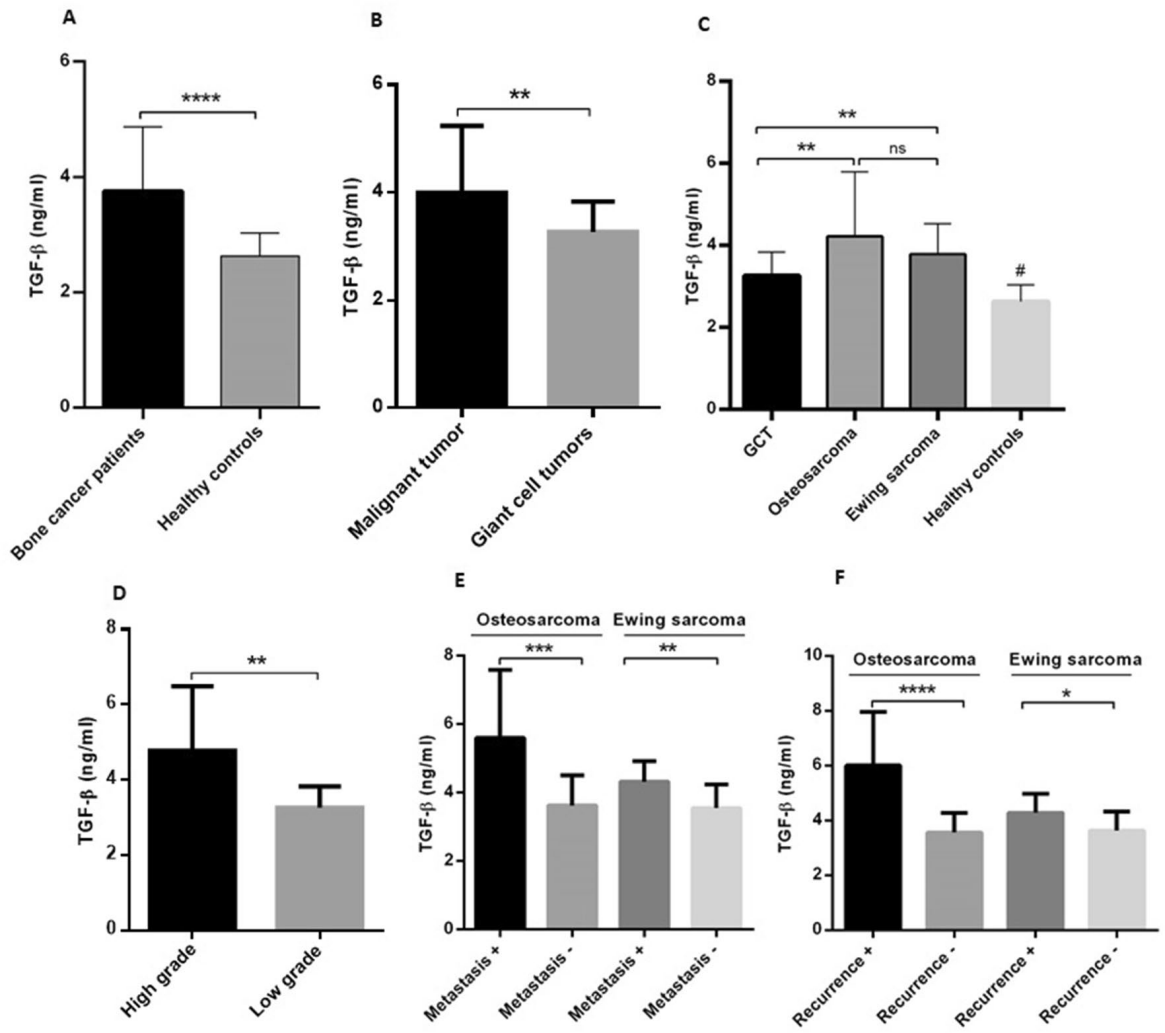
The level of TGF-β in blood of patients with primary bone tumors and healthy subjects. The TGF-β level was increased in the blood of patients with bone tumors compared to the healthy subjects (**A**) and patients with malignant tumors compared to GCT (**B**). A significant increase was observed in patients with osteosarcoma and Ewing sarcoma tumors compared to the GCT group and the increased level of TGF-β was detected in all types of tumors compared to healthy subjects (**C**). The patients with high-grade osteosarcoma tumors showed higher level of TGF-β compared to their corresponding low-grade group (**D**). The level of TGF-β in patients with metastatic (**E**) and recurrent (**F**) osteosarcoma and Ewing sarcoma tumors was increased compare to their opposite groups. The statistical differences between groups are shown as asterisks (** = *P* < 0.01, *** = *P* < 0.001, **** = *P* < 0.0001), and (ns) indicates unspecific. (#) indicates *P* < 0.0001 for comparing osteosarcoma, Ewing sarcoma, and GCT with healthy subjects.

The elevated level of TGF- β was detected in the blood of patients with primary bone tumors (3.75 ± 0.11) compared to healthy individuals (2.6 ± 0.07) (*P* < 0.0001) which is shown in Fig. 5A. Also, patients with malignant bone tumors (3.99 ± 0.16) expressed a significantly higher level of TGF- β compared to patients with GCT (3.2 ± 0.10) (*P* = 0.002) (Fig. 5B). The level of TGF- β in patients with osteosarcoma tumors (4.21 ± 0.28) was higher compared to patients with GCT (3.2 ± 0.10) (*P* = 0.002) and Ewing sarcoma tumors; while the difference between osteosarcoma and Ewing sarcoma was not considerable (Fig. 5C). It was observed that the level of TGF- β was significantly increased in patients with GCT compared to healthy subjects (*P* = 0.05); however, the elevated level of TGF- β in patients with osteosarcoma and Ewing sarcoma tumors compared to healthy subjects was more remarkable (*P* < 0.0001) (Fig. 5C). The level of TGF- β was increased significantly in patients with high-grade osteosarcoma tumors (4.77 ± 0.39) compared to their low-grade counterparts (3.25 ± 0.17) (*P* = 0.008) (Fig. 5D). The level of TGF- β was higher in patients with metastatic osteosarcoma (5.6 ± 0.66) and Ewing sarcoma tumors (4.31 ± 0.20) compared to patients with non-metastatic osteosarcoma (3.62 ± 0.19) (*P* = 0.0006) and Ewing sarcoma tumors (3.55 ± 0.14) (*P* = 0.0075) (Fig. 5E). Also, the level of TGF- β was higher in patients with recurrent osteosarcoma (6 ± 0.68) and Ewing sarcoma tumors (4.26 ± 0.26) compared to patients with non-recurrent osteosarcoma (3.56 ± 0.15) (*P* < 0.0001) and Ewing sarcoma tumors (3.62 ± 0.14) (*P* = 0.04) (Fig. 5F).

### The association of PD-L1, TGF- β and IFN-γ with different demographic features of patients with osteosarcoma, Ewing sarcoma and GCT and their diagnostic values

The correlation of PD-L1 gene and protein, TGF- β and IFN-γ levels with patients' demographic demographic (Age) and pathologic (metastasis and recurrence status and malignancy) features was evaluated. Based on data (Table 1), among the examined factors, only the value of IFN-γ showed a significant correlation with the age of the patients (*P* = 0.044). However, tumor size and tumor malignancy was significantly correlated with PD-L1 gene and protein, TGF- β and IFN-γ levels in this study (*P* = 0.001). Also, positive association was observed between PD-L1 gene and protein, TGF- β and IFN-γ levels and metastasis and recurrence (*P* = 0.0001). Based on data, PD-L1 gene and protein and TGF- β and IFN-γ levels were significantly correlated together (*P* = 0.001). In addition, the logistic regression analysis showed that the IFN-γ (*P* = 0.002) and PD-L1 (*P* = 0.0001) protein level can predict the bone tumor malignancy significantly (Table 2). Also the PDL-1 protein (*P* = 0.0001) and TGF- β (*P* = 0.04) and IFN-γ (*P* = 0.005) levels could predict the bone tumor grade significantly. The effective impact of TGF- β (*P* = 0.0001) and PD-L1 gene (*P* = 0.001) and protein (*P* = 0.007) in predicting tumor metastasis was remarkable in bone tumors. Also, our data showed that PD-L1 gene (*P* = 0.02) and protein (*P* = 0.016) and TGF-β (*P* = 0.001) and IFN-γ (*P* = 0.03) levels could predict bone tumor recurrence effectively. Additionally, the ROC curve analysis was applied to evaluate whether the PD-L1 gene and protein and TGF- β and IFN-γ levels can discriminate different patient groups and tumor subtypes and our data showed that the abovementioned factors could distinguish patients with bone tumors and healthy individuals, also malignant and non-cancerous tissues, GCT and non-cancerous tissues and malignant and GCT, significantly. The details of analysis and cut-off values, Area under the curve (AUC), sensitivity and specificity also P values are summarized in supplementary table 1 and the ROC curves of PD-L1 gene levels between different groups are shown in Supplementary Fig. 1A–D, the ROC curves of PDL-1 protein levels between different groups are shown in Supplementary Fig. 1E–H, the ROC curves of IFN-γ levels between different groups are shown in Supplementary Fig. 2A–D and the ROC curves of TGF- β levels between different groups are shown in Supplementary Fig. 2E–H.

**Table 1:** The association of PDL-1, INF-γ and TGF-β with bone cancer different features.

Variable	Serum		Tumor (Gene expression)	Tumor (Protein expression)
	TGF-β	INF-γ	PDL-1	PDL-1
Age				
Correlation	0.031	0.214	0.056	− 0.022
*P* value	0.774	0.044*	0.600	0.838
Tumor size				
Correlation	0.490	0.468	0.493	0.631
*P* value	0.0001**	0.001**	0.0001**	0.001**
Malignancy				
Correlation	0.310**	0.439**	0.042**	0.698**
*P* value	0.003	0.000	0.0001	0.0001
Metastasis				
Correlation	0.543**	0.450**	0.653**	0.652**
*P* value	0.0001	0.0001	0.0001	0.0001
Recurrence				
Correlation	0.582**	0.476**	0.512**	0.505**
*P* value	0.0001	0.0001	0.0001	0.0001
TGF-β				
Correlation	1.000	0.501**	0.368**	0.402**
*P* value		0.0001	0.0001	0.0001
INF-γ				
Correlation	0.501**	1.000	0.353**	0.581**
*P* value	0.0001		0.001	0.0001
PDL-1 gene				
Correlation	0.368**	0.353**	1.000	0.568**
*P* value	0.0001	0.001		0.0001
PDL-1 protein				
Correlation	0.402**	0.581**	0.568**	1.000
*P* value	0.0001	0.0001	0.0001	

The statistical differences between groups are shown as asterisks (*= *P* < 0.05, **= *P* < 0.01).

**Table 2:** The regression of PDL-1, INF-γ and TGF-β (Logistic regression).

Dependent	Independent variable	OR	95% CI	*P* value
Malignancy (GCT Vs. Malignant)	TGF-β	1.86	0.85–4.09	0.120
	INF-γ	1.17	1.06–1.29	0.002
	PDL-1 (Gene)	2.78	0.38–20.51	0.317
	PDL-1 (Protein)	1.37	1.20–1.57	0.0001
Metastasis (Negative Vs. Positive)	TGF-β	6.22	2.05–18.8	0.0001
	INF-γ	1.28	1.24–16.2	0.48
	PDL-1 (Gene)	1.21	1.10–1.33	0.001
	PDL-1 (Protein)	1.40	1.09–1.78	0.007
Recurrence (Negative Vs. Positive)	TGF-β	11.04	2.63–46.4	0.001
	INF-γ	11.74	5.06–24.24	0.03
	PDL-1 (Gene)	10.77	1.44–80.23	0.02
	PDL-1 (Protein)	1.12	1.02–1.24	0.016

### The CD4/CD8 ratio decreased in patients with osteosarcoma tumors

**Figure 6:**
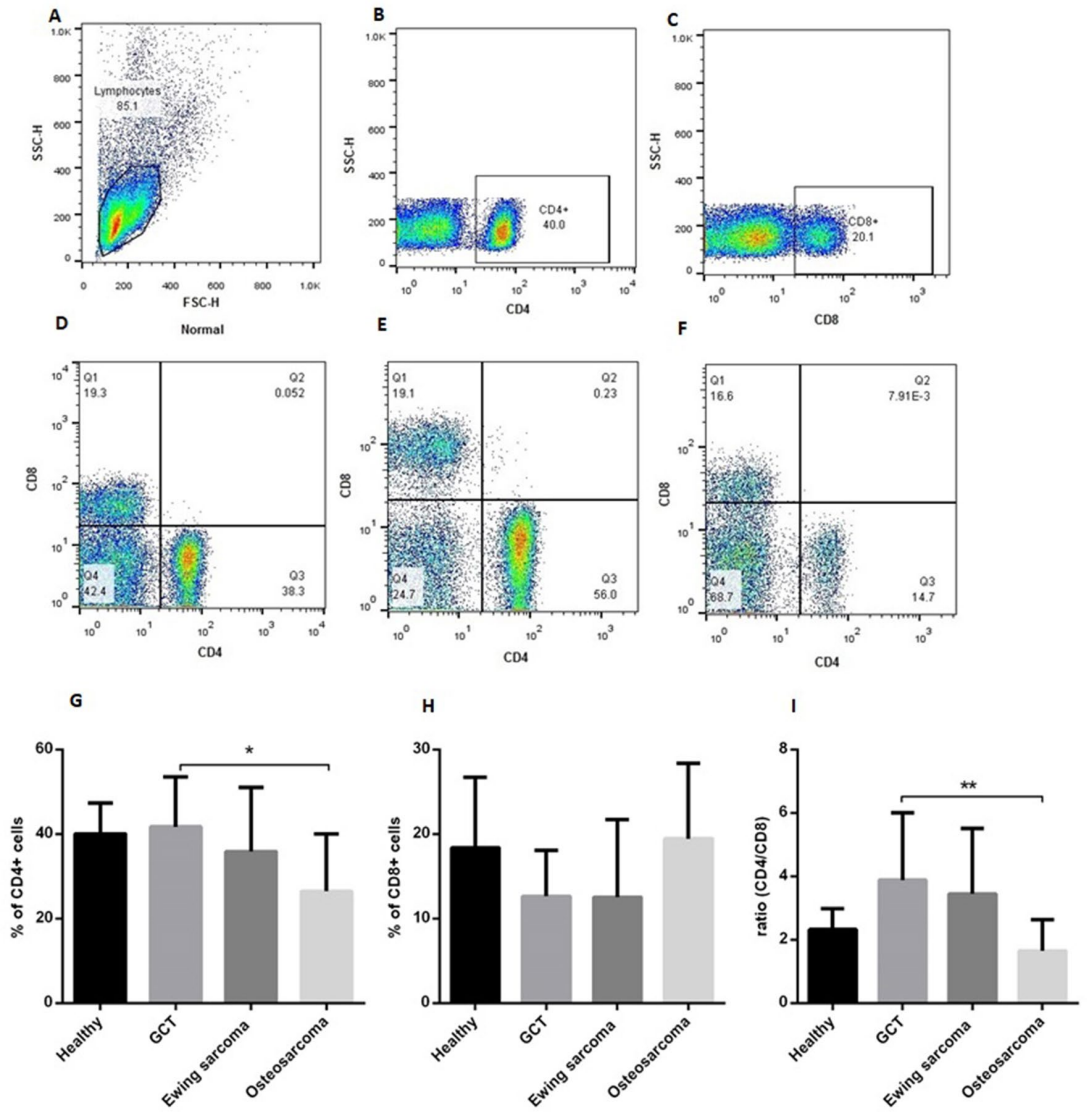
The CD4/CD8 ratio in patients with primary bone tumors. The pattern of CD4^+^ and CD8^+^ T cells in patients with different primary bone tumors is shown. The flow cytometry plots indicate the total events (**A**), CD4^+^-Gated events (**B**), CD8^+^-Gated events (**C**) CD4^+^/CD8^+^ cells in healthy subject (**D**), CD4^+^/CD8^+^ cells in patient with GCT (**E**), CD4^+^/CD8^+^ cells in in patient with malignant tumor (**F**). Also (**G**) represents percentages of CD4^+^ cells in different groups, (**H**) represents percentages of CD8^+^ cells in different groups and (**I**) represents the ratio of CD4/CD8 in heathy and patients. The statistical differences between groups were analyzed and are shown as asterisk on the bars (* = *P* < 0.05, ** = *P* < 0.01).

The possible changes in the shift of T helper cells and the pattern of CD4 + and CD8 + T cells in patients with different primary bone tumors were evaluated by determining the percentages of CD4 + and CD8 + T cells using flow cytometry. The flow cytometry histograms of the total events, gating strategy, and representative results of CD4 + /CD8 + cells in healthy subjects and patients are shown in Fig. 6A–F. Based on the data, the percentage of CD4 + cells were decreased in patients with osteosarcoma tumors compared to patients with GCT (*P* = 0.017) and healthy subjects (not significant statistically) (Fig. 6G). Despite the relative increase in the percentage of CD8 + cells in patients with osteosarcoma, the percentage of these cells did not show a significant change in any of the tumor groups compared to healthy individuals (Fig. 6H). However, the CD4 + /CD8 + ratio was decreased significantly in patients with osteosarcoma compared to GCT tumors (*P* = 0.01) (Fig. 6I).

## Discussion

The adaptive immune mechanism is a strategy used by tumor cells to escape from the immune system and immune surveillance and understanding the molecular mechanism involved in this process can provide new insights for controlling the growth and progression of tumor cells^15^. Recent reports demonstrated that increased tumor-associated PD-L1 levels are correlated with the activation of epithelial-mesenchymal transition (EMT)-related signaling molecules required for tumorigenesis and metastasis^24^. It is well-documented that activation of pro-survival and oncogenic signaling mediators and pathways such as Mitogen‑activated protein kinase (MAPK)^25^, PI3K-AKT pathway^26^, JAK/STAT pathway^25^ and NF-ĸB transcription factor are involved in up-regulation and abundance of PD-L1 that facilitate tumor cell proliferation and growth^15^. Increased expression of PD-L1 has been demonstrated in several types of cancers that were associated with poor prognosis^27^. However, the study of PD-L1, as an immune checkpoint, and possible factors affecting its regulation in primary bone tumors has been limited. Evaluating the expression pattern of PD-L1 in primary bone tumors in the current study revealed an increase in the gene and protein level of PD-L1 in osteosarcoma, Ewing sarcoma and GCT tissues compared to non-cancerous bone tissues. The higher level of PD-L1 in osteosarcoma and Ewing sarcoma, as malignant tumors, compared to GCT, as intermediate tumors, indicates the effective role of PD-L1 in tumor proliferation and deterioration. In addition, an elevated level of the PD-L1 gene and protein in metastatic and recurrent osteosarcoma and Ewing sarcoma tumors, strengthen the hypothesis of the possible role of PD-L1 in the escape of tumor cells from the immune system and increasing their proliferative invasion power. In support of our data, it was shown that PD-L1 mRNA level was increased in osteosarcoma tissues and its over-expression induced osteosarcoma K7M2 cell proliferation and immune escape ability^14^. Also, a correlation of PD-L1 expression with poor overall survival of patients with sarcomas, suggests it as a possible predicative biomarker for bone tumor prognosis^16^. Additionally, the PD-L1 protein expression was detected in tumor tissues of patients with Ewing sarcoma that revealed higher level of expression in metastatic tumors that showed no relation to the prognosis^28^. On the other hand, it was shown that blockage of IFN-γ receptor in Ovarian cancer cells resulted in reduction of PD-L1 expression and induction of CD8^+^ T-cell infiltration in to the site of tumor which indicating that IFN-γ-induced PD-L1 expression may play decisive role in cancer cell immune escape^29^. To clarify the possible effect of IFN-γ on the PD-L1 expression, the expression level of IFN-γ was evaluated in patients and our data revealed higher level of IFN-γ in patients with bone tumors. The IFN-γ level was more prominent in patients with osteosarcoma compared to GCT and Ewing sarcoma and the association of IFN-γ level with tumor metastasis, grade and recurrence was detected in osteosarcoma patients. Evaluation of IFN-γ levels in patients with bone tumors has been limited, but a mechanistic study suggests that IFN-γ induced the growth of HOS-Y1 osteosarcoma cells through activation of growth factors^30^. It seems that both pro and anti-tumorigenic effects are regulated by IFN-γ in tumor microenvironment depending on the type of mediators that are expressed along with it^21^ and its stimulating role in increasing the level of immune escape checkpoints such as PD-L1 can strengthen the hypothesis of its tumorigenic effects. Although, changes in IFN-γ levels in metastatic and recurrent Ewing tumors were not considerable, our results are generally in favor of this hypothesis and needs to be expanded by further mechanistic studies. Notably, the implication of TGF- β in immune system homeostasis has been extensively studied and it is postulated that over-expression of TGF- β in cancer is associated with suppression of cytotoxic T cells (CTL) and immunosuppressive immune mediators such as regulatory T cells (Tregs) leading to immune suppression^23^. Interestingly, it was shown that administration of bintrafusp alfa as a bi-functional agent capable of inhibiting PD-L1 and TGF- β simultaneously, leading to TGF- β trap in tumor microenvironment and EMT prevention in carcinoma cells^31^. Investigating the TGF- β expression profile in the current study revealed that TGF- βl level increased in patients with bone tumors compared to healthy controls and the elevated level of TGF- β was prominent in osteosarcoma and Ewing sarcoma compared to GCT. The association of TGF- β elevation with tumor metastasis and recurrence in patients with osteosarcoma and Ewing sarcoma can further confirm the pro-tumoral effect of TGF- β in bone. Also the simultaneous over-expression of TGF- β and PD-L1 with the same pattern in our samples reinforce the possible role of TGF- β in inhibiting immune surveillance through PD-L1. Accordingly, it was shown that TGF- β is effective in bone angiogenesis and remodeling leading to bone cell migration and proliferation^32^. Amongst the TGF- β isoforms, TGF- β1 that is surveyed in the current study, is directly involved in bone remodeling and favors bone formation as well as bone resorption and destruction in a dose-dependent manner^33^. The relationship between TGF- β1 and EMT process which is essential for tumor cell progression and migration can be explained through the stimulatory effect of TGF- β1 on matrix metalloproteinase (MMP) such as MMP-2 and MMP-9^34^. In support of this evidence, our previously published results demonstrated the elevation of MMP-9 gene and protein in osteosarcoma and Ewing sarcoma tumors that was associated with tumor recurrence and metastasis^35^. The effect of TGF- β on cancer progression might be explained by the regulatory effect of TGF- β on T cell proliferation since it was shown that TGF- β inhibits T cell proliferation and function^36^. Scattered piece of evidence showed the involvement of CD + 4 and CD + 8 cells in osteosarcoma microenvironment that might be implicated in tumor development^37^. Accordingly, Hashimoto et al., showed that CD4^+^ and CD8^+^ cells were co-expressed with PDL-1 in osteosarcoma tumor sites and seemed to be involved in tumor pathogenesis^38^. Our data revealed a significant decrease in the ratio of CD4^+^/CD8^+^ also the percentage of CD4^+^ cells in peripheral blood of patients with osteosarcoma compared to patients with GCT and healthy controls. It is taken for granted that low CD4^+^/CD8^+^ reflects T cell exhaustion and lower ability of immune system to response accurately that is rationally expected in patients with cancer^39^ and our data is in line with this fact; however, to determine the exact role of TGF- β on the regulation of the T cell differentiation, it is necessary to investigate CD4^+^ and CD8^+^ lymphocytes in the tumor site and pharmacologically targeting TGF- β in cancer cells that will be surveyed in future study. In the current study PD-L1 gene and protein, TGF- β and IFN-γ were correlated significantly together indicating the possible cross talk between these pathways in regulating bone tumor fate. Also it seemed that PD-L1, TGF- β and IFN-γ has diagnostic value to discriminate primary bone tumors and tumor sever subtypes that requires to be more comprehensively studies in future.

## Conclusion

**Figure 7:**
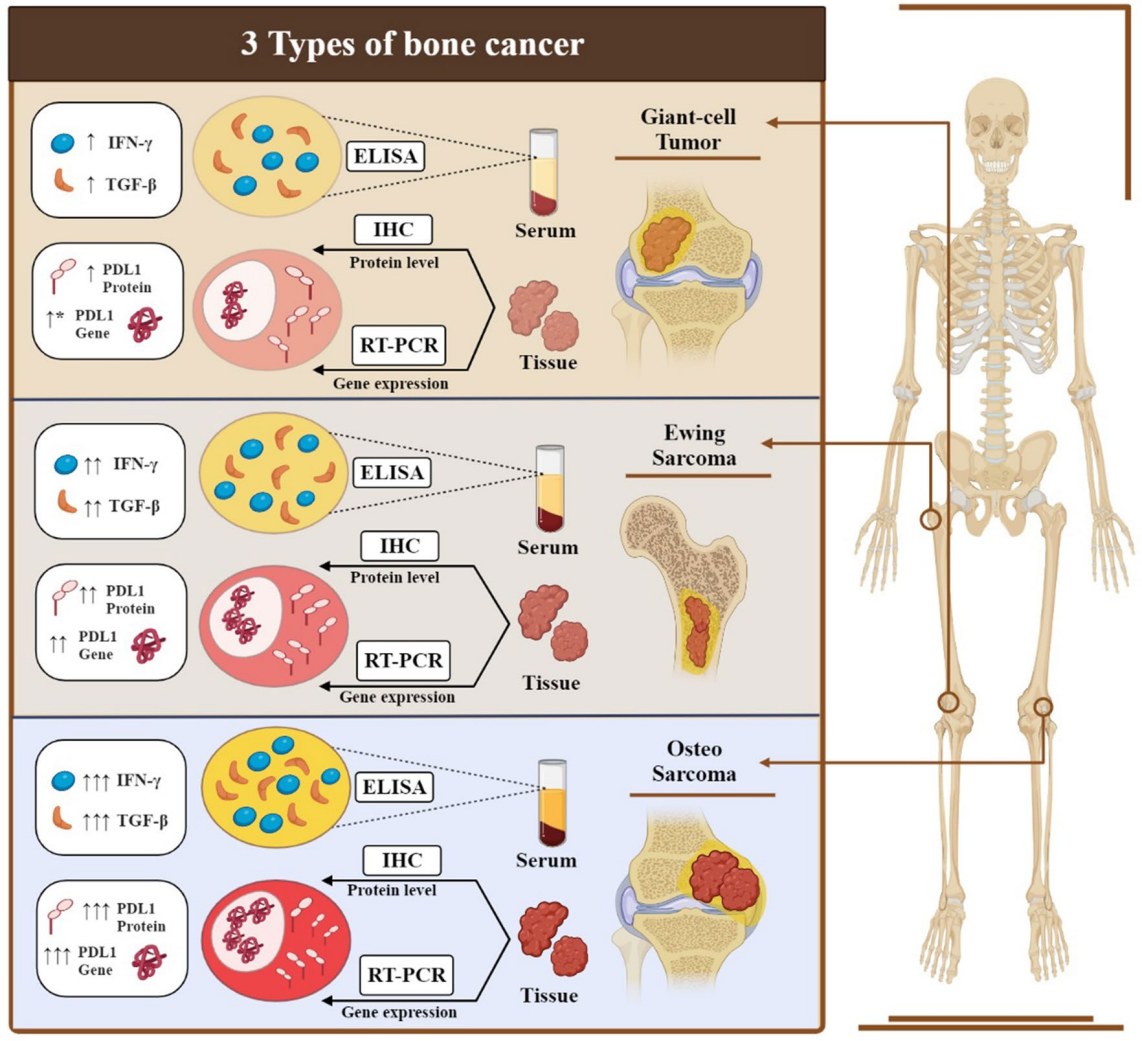
The alteration in the expression profile of PD-L1, TGF-β, IFN-γ in bone tumors. Three types of bone tumors, namely GCT, Ewing sarcoma, and osteosarcoma, are depicted schematically to illustrate the changes in PDL-1 gene and protein expression, as well as serum levels of TGF-β and IFN-γ.

Our results validated pervious evidences regarding the possible photogenic role of PD-L1 in bone tumors, however inclusion of assessing another regulator such as TGF- β and IFN-γ as well as investigating of other bone sarcomas with higher tumor diversity and malignancy allowed us to have more comprehensive picture of how PD-L1 may work in these tumors (Fig. 7). Our data provides insights into the possible effect of TGF- β and IFN-γ as secretory actors in microenvironment in PD-L1 -induced bone tumor pathogenesis and provides impetus to study more. These data may shed light on the potential benefits of combing anti- PD-L1-anti-TGF-β immunotherapy and traditional therapeutic strategies to increase the effectiveness of common treatments and eliminate defects for those patients with over-expression of PD-L1.

## Materials and methods

### Patients and sample collection

A total number of 180 bone tissues including 30 giant cell tumors (GCT) and 30 paired-tumor margin tissues, 30 osteosarcoma tumors and 30 paired-tumor margin tissues, 30 Ewing sarcoma tumors and 30 paired noncancerous tissues were collected and enrolled in the current study. According to the 2020 WHO Classification of bone Tumors, osteosarcoma tumors are a type of osteogenic tumor that develop in long bones, mainly in the distal femur and tibia. Similarly, Giant Cell Tumor is a type of bone tumor that is locally aggressive and rarely spreads to other parts of the body, consisting of giant cells surrounded by mononuclear stromal cells. Additionally, Ewing Sarcoma is a rare type of sarcoma characterized by round cells and EWSR1-ETS fusions, typically occurring in long bones^7,40^. Also, a total number of 90 blood samples including 30 samples from patients with osteosarcoma, 30 samples from patients with Ewing sarcoma, 30 blood samples from patients with GCT, and 30 blood samples from healthy individuals were collected and applied for serum and peripheral blood mononuclear cell (PBMC) separation. The healthy subjects were matched as a matter of age and gender with the patients. The sample collection and all relevant assessments were conducted based on the declaration of Helsinki^41^ also the study was approved ethically by the ethics committee of the Iran University of Medical Sciences and patients were informed about the study process and consent was obtained based on ethical standards in research. The study is performed on a previously collected cohort of bone samples and the patient’s demographic characteristics are described in our previous study^35^. Tumor and non-cancerous tumor margin tissues were collected freshly in a wide surgery of patients who were undergoing treatment and surgery at Shafa Orthopedic Hospital. The blood samples were taken immediately before surgery in a fasting condition from each patient. The process of tissue collection and preservation is according to the protocol mentioned in our previous studies^35,42^. In the current study, patients with high-grade osteosarcoma indicating High-grade surface osteosarcoma and low-grade osteosarcoma indicating low-grade central osteosarcoma were participated and categorized and considered respectively^40^. As all Ewing sarcomas were of high grade and all GCTs were of intermediate grade based on the 2020 WHO Classification of Tumors of Bone^40^, only osteosarcoma tumors were analyzed in the study to assess the relationship between grade and the factors under investigation. Moreover, based on the pathology reports if there was evidence of distant metastasis to other organs, that sample was included in the category of metastatic tumors. Accordingly, both metastatic and non-metastatic bone tumors were studied in this survey and primary tumors of the bone were collected in both groups. Also, if the tumor relapse was occurred following the end of the patient's treatment period, that tumor was categorized into the recurrent tumor group and both recurrent tumors and non-recurrent tumors were collected and studied in this survey. As reported previously^35^, the average age of patients with osteosarcoma, Ewing sarcoma and GCT was 37.76 ± 3.17, 25.60 ± 1.64 and 31.50 ± 2.8, respectively. Also, 53.3%, 40% and 56.7% of patients with osteosarcoma, Ewing sarcoma and GCT were male, respectively. Moreover, 63.3% and 66.7% of osteosarcoma and Ewing sarcoma tumors were categorized as high-grade, also 30% of osteosarcoma and Ewing sarcoma tumors were metastatic. Additionally, 26.7% of osteosarcoma tumors and 23.3% of Ewing sarcoma tumors were recurrent.

### RNA extraction, cDNA synthesis, and Real-Time PCR

The protocol for examining gene expression and related processes was carried out in accordance with our previous studies^42,43^. Briefly, the total RNA was extracted from all tissue samples using Trizol (Invitrogen, Grand Island, USA) and phenol–chloroform protocol. The quantity of the extracted RNA from each tissue was assessed using the Nanodrop spectrophotometer (Nanodrop Technologies) and the quality and integrity of the extracted RNAs were evaluated using electrophoresis (with 1% agarose gel). For each tissue, 1 µg RNA was used to synthesize cDNA using the PrimeScript First Strand cDNA Synthesis Kit (Takara, Japan) and the PDL-1 mRNA level was measured using SYBR Premix Ex Taq II (Takara, Japan) by Applied Biosystems Step One Plus, Real-Time System (Applied Biosystems, USA). The β-actin level of expression was measured for all samples as an endogenous control and the quality of PCR products was controlled using electrophoresis. The primer sequences for PDL-1 and β-actin were as follows: PD-L1 forward primer: 5′– TCATCCCAGAACTACCTCTG -3′, PD-L1 reverse primer: 5′- CGGAAGATGAATGTCAGTGC -3′ (Tm = 58), β-actin forward primer: 5’-GAT CTC CTT CTG CAT CCT GT-3', β-actin reverse primer: 5′-TGG GCA TCC ACG AAA CTA C- 3′ (Tm = 57) and the melt curve of primers were considered as primer specificity for each sample. The PCR reaction was implemented as 10 min at 95 °C (Holding stage), 40 cycles of 15 s at 95 °C, 20 s at 55 °C, and 35 s at 60 °C (Cycling stage) and 15 s at 95 °C, 30 s in 65 °C, and 15 s at 95 °C (Melt curve stage). The comparative Ct (2^−ΔCt^) method was used to analyze the PD-L1 gene expression level between samples, where ΔCt is obtained by subtracting the Ct of PD-L1 from the Ct of β-actin which was calculated separately for each sample.

### Assessment of IFN-γ and TGF- β

The circulating level of IFN-γ and TGF- β in the serum of patients with different types of primary bone tumors was assessed using ELISA assay kits. The Human IFN-γ (R&D, USA, Cat No.# DY285) and the Human TGF- β (R&D, USA, Cat No.# DY240) was applied according to the manufacturer's instructions. Briefly, following plate preparation, 100 mL of sample or standard solutions were added to the dilution reagent in each well of the plate and incubated for 2 h at room temperature, following appropriate washing, 100 µl of the detection antibody diluted in the dilution reagent was added to each well and incubated. Following washing and aspiration, 100 µl of the required dilution of streptavidin HRP was added to each well and incubated for 20 min at room temperature in a dark condition. Wells were washed and 100 µl of substrate solution was added to each well and incubated for 20 min at room temperature in a dark condition. The amount of 50 µL of stop solution was added to the wells and the optical density was determined at 450 nm using a microplate reader. The analytical sensitivity for Human IFN-γ kit was 15.6 pg/ml and for the Human TGF- β kit was 31.2 pg/ml.

### PD-L1 protein assessment via immunohistochemistry

The protein level of PD-L1 was measured in tumor and non-cancerous tissues of patients with bone tumors using immunohistochemistry. The anti-PD-L1 antibody (Abcam, USA, Cat No. # ab233482) and the anti-rabbit IgG HRP-conjugated secondary antibody (Cell Signaling, the Netherlands, Cat No. # 7074) were used for staining with the dilution of 1:200 for both antibodies. The basis of immunohistochemistry and its related processes are in accordance with our previous studies on these samples^35^. Briefly, the Optimal Cutting Temperature (OCT) embedding medium was applied to prepare tissue blocks and paraformaldehyde (4%) was used for tissue fixation. The cryotome was used for sectioning tissues (10 nm) and Triton (3%) was used to increase the membrane permeability of the tissues for proper staining. The goat serum (10%) was used for blocking the non-specific antigenic sites. To visualize the horseradish peroxidase activity, 1 µl of 3′-diaminobenzidine (DAB) chromogen and its substrate was applied. The intensity of PD-L1 staining was scored by two separate pathologists and the percentage of positive reactivity was quantified using Image J software based on the analysis protocol that was described previously^44,45^. The representative images indicating weak intensity (< 10% immune reactivity), moderate intensity (10–20% immune reactivity) and strong intensity (> 20% immune reactivity) of staining are provided in Fig. 3.

### CD4^+^ and CD8^+^ quantification in blood of patients with primary bone tumors

The percentages of T cells were assessed by flow cytometry according to the manufacturer's protocol. Briefly, 10 ml blood from each patient and healthy controls was collected and subjected to the peripheral blood mononuclear cells (PBMC) separation using the density gradient centrifugation by Ficoll-Hypaque (Sigma Chemical Co, St Louis, MO, USA). The cells were counted using a hemocytometer and the amount of 2 × 10^5^ of cells for each sample were stained with CD4^+^ antibody labeled with FITC (fluorescein isothiocyanate) and CD8^+^ antibody labeled with PE (phycoerythrin) and incubated for an appropriate time and the percentages of CD4^+^ and CD8^+^ cells for each sample was measured using a FACS Calibur flow cytometer (Becton Dickinson, SanJose, USA) and data analyzed with the supplied software.

### Statistical analysis

To evaluate the normal distribution of data, the Kolmogorov–Smirnov analysis was applied. Based on the results, a nonparametric Mann–Whitney U-test was used to analyze the PD-L1 gene expression level and PD-L1 protein level between tumors and non-cancerous tissues also between different tumor types and their subtypes (Results are presented in Figs. 1 and 2). The parametric unpaired t-test was used to analyze the IFN-γ and TGF- β levels between patients and healthy subjects and also between patients with different tumor types (Results are shown in Figs. 4 and 5). The comparison between the group of patients and healthy people as well as different groups of patients in terms of the percentage of CD4^+^ and CD8^+^ cells was investigated using an unpaired t-test (Results are illustrated in Fig. 6). The receiver operating characteristic (ROC) curve was applied to determine the diagnostic value of the PD-L1 gene and protein level to discriminate patients form healthy controls as well as patients with different tumor types. The area under the curve (AUC) and the optimal cut-off values points were determined for each group based on Youden index^46^ (Graphs are shown in supplementary Figs. 1 and 2 and supplementary Table 1).The Spearman's correlation coefficient test was applied to determine the association between the PD-L1 gene and protein, IFN-γ and TGF- β levels with the patient’s demographic and pathologic features (Results are presented in Table 1). The possibility of PD-L1 gene and protein, IFN-γ and TGF- β levels to predict the patient’s tumor features were calculated using logistic regression (Results are shown in Table 2). The Graph Pad Prism Version 6 (Graph Pad Software, San Diego California) and Statistical Package for Social Science (SPSS v.16) was applied for statistical analysis, and the *P*-value < 0.05 (two-tailed) was considered statistically significant.

### Ethics approval and consent to participate

The project was approved ethically by ethics committee of the Vice president of research of Iran University of Medical Sciences with ethics committee code: IR.IUMS.FMD.REC.1400.411. The project process was implemented based on the Declaration of Helsinki and its related standards, protocols, guidelines and regulations^47^. All patients and healthy subjects were informed about the project before enrolled in the study and the informed consent to participate in the project was seen and signed by all of the participants.

### Supplementary Information

Supplementary Legends.

Supplementary Table 1.

Supplementary Figure 1.

Supplementary Figure 2.

## Data Availability

All data generated or analyzed during this study are included in the manuscript. The raw data can be provided by the corresponding author on reasonable request.

## Abbreviations

PD-1: Programmed death 1
PD-L1: Programmed death ligand1
NK cells: Natural killer cells
IFN-γ: Interferon γ
PKD2: Protein kinase D isoform 2
JAK: Janus kinase
STAT: Signal transducer and activator of transcription
TGF-β: Growth factor-beta
Tregs: Regulatory T cells
GCT: Giant cell tumors
PBMC: Peripheral blood mononuclear cell
OCT: Optimal Cutting Temperature
DAB: 3′-Diaminobenzidine
ROC: Receiver operating characteristic
AUC: Area under the curve
SEM: Mean and standard error of mean
H&E: Hematoxylin and eosin
EMT: Epithelial-mesenchymal transition
MAPK: Mitogen‑activated protein kinase
CTL: Cytotoxic T cells
Tregs: Regulatory T cells
MMP: Matrix metalloproteinase
