# Supplementary material for: The expression changes of PD-L1 and immune response mediators are related to the severity of primary bone tumors
Source: Sci Rep. 2023 Nov 22;13:20474. doi: 10.1038/s41598-023-47996-8 (PMC10665336; doi:10.1038/s41598-023-47996-8)
Supplement: Supplementary file 1 — Supplementary Legends. [file 41598_2023_47996_MOESM1_ESM.docx]

**Supplementary figure legends:**

**Supplementary figure 1: The ROC Curves of PD-L1 gene and protein levels between different tumor groups**

The diagnostic values of PD-L1 gene and protein expression levels between different patient’s groups are calculated using ROC curve analysis. A: indicates ROC curve of PD-L1 gene expression between bone tumors and normal tumor margins; B: indicates ROC curve of PD-L1 gene expression between malignant tumor group and normal tumor margins; C: indicates ROC curve of PD-L1 gene expression between GCT group and normal tumor margins; D: indicates ROC curve of PD-L1 gene expression between malignant and GCT bone tumor groups; E: indicates ROC curve of PD-L1 protein level between bone tumors and normal tumor margins; F: indicates ROC curve of PD-L1 protein level between malignant tumor group and normal tumor margins; G: indicates ROC curve of PD-L1 protein level between GCT group and normal tumor margins; H: indicates ROC curve of PD-L1 protein level between malignant and GCT groups.

**Supplementary figure 2: The ROC Curves of IFN-γ and TGF- β** **levels between different patient’s groups**

The diagnostic values of IFN-γ and TGF- β levels between different patient’s groups are assessed using ROC curve analysis. A: indicates ROC curve of IFN-γ level between patient and healthy control groups; B: indicates ROC curve of IFN-γ level between patient’s with malignant tumor and healthy control groups; C: indicates ROC curve of IFN-γ level between patient’s with GCT = and healthy control groups; D: indicates ROC curve of IFN-γ level between patient’s with malignant and GCT groups; E: indicates ROC curve of TGF- β level between patient and healthy control groups; F: indicates ROC curve of TGF- β level between patient’s with malignant tumor and healthy control groups; G: indicates ROC curve of TGF- β level between patient’s with GCT and healthy control groups; H: indicates ROC curve of TGF- β level between patient’s with malignant and GCT groups.
