# Supplementary material for: The expression changes of PD-L1 and immune response mediators are related to the severity of primary bone tumors
Source: Sci Rep. 2023 Nov 22;13:20474. doi: 10.1038/s41598-023-47996-8 (PMC10665336; doi:10.1038/s41598-023-47996-8)
Supplement: Supplementary file 2 — Supplementary Table 1. [file 41598_2023_47996_MOESM2_ESM.docx]

**Supplementary table 1: The value of PDL-1, INF-γ and TGF-β to discriminate between different groups of primary bone tumors (ROC curve information)**

| **Variable** | **Groups** | **Cutoff point** | **Sensitivity (%)** | **Specificity (%)** | **AUC** | **P-value** |
| --- | --- | --- | --- | --- | --- | --- |
| **INF-γ** | Patient Vs. Control | < 0.25 | 100 | 88 | 0.96 | 0.0001 |
| **INF-γ** | Malignant Vs. Control | < 0.26 | 100 | 97 | 0.99 | 0.0001 |
| **INF-γ** | GCT Vs. Control | < 0.23 | 100 | 70 | 0.92 | 0.0001 |
| **INF-γ** | Malignant Vs. GCT | < 0.43 | 87 | 68 | 0.81 | 0.0001 |
| **TGF-β** | Patient Vs. Control | < 3.18 | 100 | 69 | 0.88 | 0.0001 |
| **TGF-β** | Malignant Vs. Control | < 3.18 | 100 | 78 | 0.91 | 0.0001 |
| **TGF-β** | GCT Vs. Control | < 2.86 | 83 | 70 | 0.81 | 0.0001 |
| **TGF-β** | Malignant Vs. GCT | < 3.59 | 73 | 62 | 0.7 | 0.0013 |
| **PDL-1**  **(Gene)** | Patient Vs. Normal margin | < 0.11 | 81 | 96 | 0.93 | 0.0001 |
| **PDL-1**  **(Gene)** | Malignant Vs. Normal margin | < 0.15 | 82 | 100 | 0.95 | 0.0001 |
| **PDL-1**  **(Gene)** | GCT Vs. Normal margin | < 0.10 | 90 | 90 | 0.92 | 0.0001 |
| **PDL-1**  **(Gene)** | Malignant Vs. GCT | < 0.56 | 77 | 70 | 0.77 | 0.0001 |
| **PDL-1**  **(Protein)** | Patient Vs. Control | < 15.87 | 100 | 87 | 0.97 | 0.0001 |
| **PDL-1**  **(Protein)** | Malignant Vs. Control | < 17.41 | 100 | 100 | 1 | 0.0001 |
| **PDL-1**  **(Protein)** | GCT Vs. Control | < 12.89 | 87 | 83 | 0.90 | 0.0001 |
| **PDL-1**  **(Protein)** | Malignant Vs. GCT | <21.05 | 80 | 98 | 0.93 | 0.0001 |
