# Supplementary figures and images for: The expression changes of PD-L1 and immune response mediators are related to the severity of primary bone tumors
Source: Sci Rep. 2023 Nov 22;13:20474. doi: 10.1038/s41598-023-47996-8 (PMC10665336; doi:10.1038/s41598-023-47996-8)

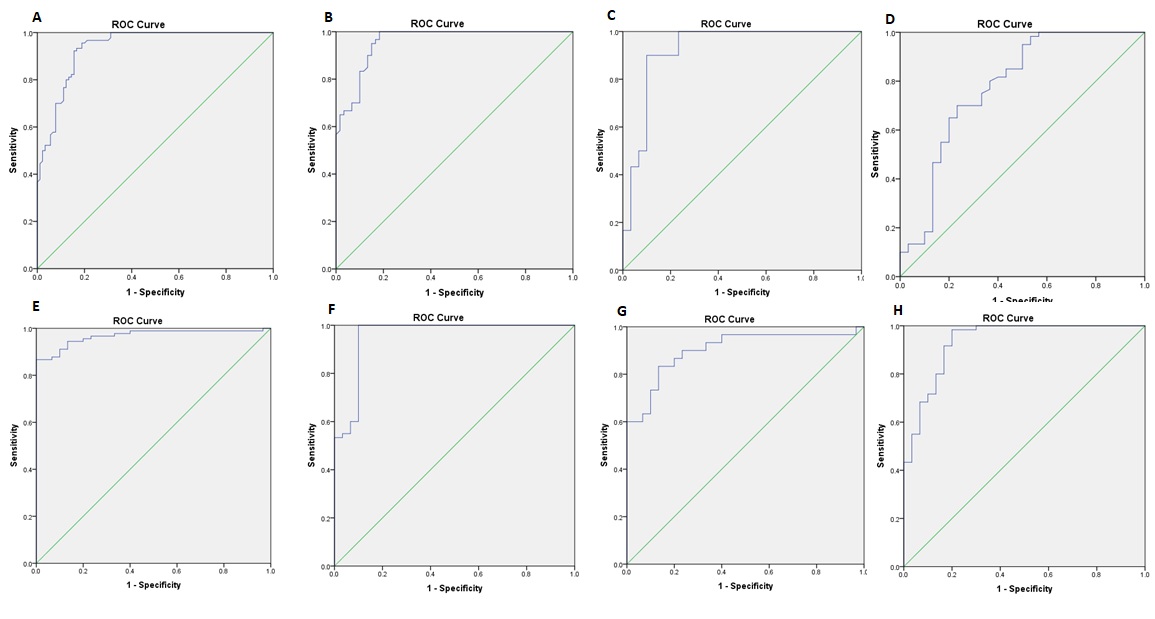

Supplement: Supplementary file 3 — Supplementary Figure 1. [file 41598_2023_47996_MOESM3_ESM.jpg]

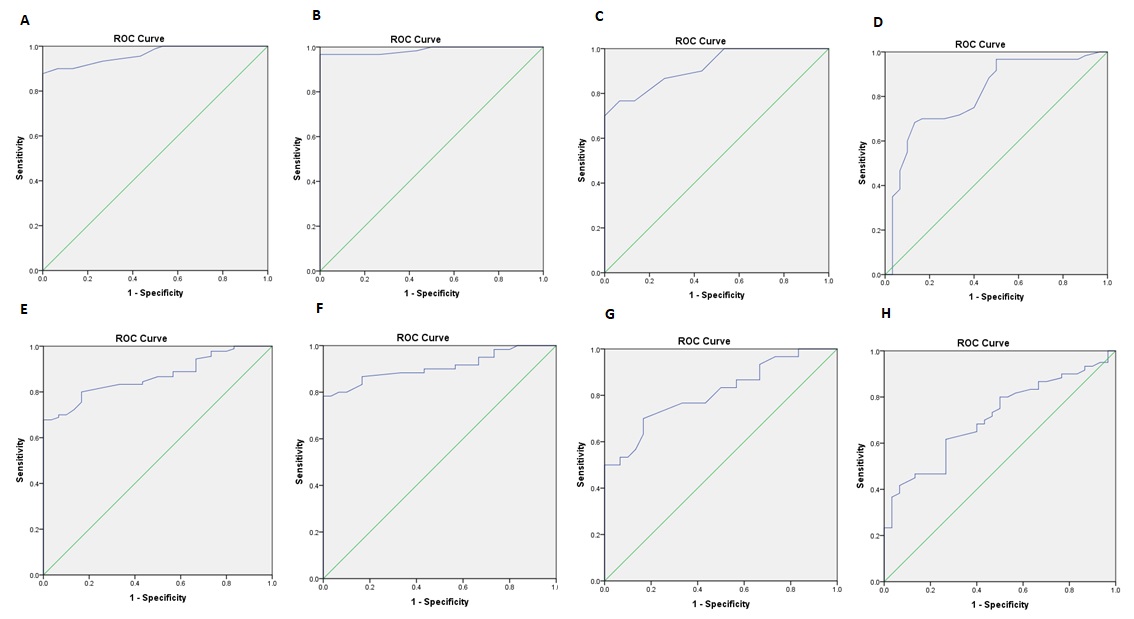

Supplement: Supplementary file 4 — Supplementary Figure 2. [file 41598_2023_47996_MOESM4_ESM.jpg]
